# Methodological and ethical challenges in studying patients’ perceptions of coercion: a systematic mixed studies review

**DOI:** 10.1186/1471-244X-14-162

**Published:** 2014-06-04

**Authors:** Päivi Soininen, Hanna Putkonen, Grigori Joffe, Jyrki Korkeila, Maritta Välimäki

**Affiliations:** 1 Department of Nursing Science, University of Turku, Turku, Finland; 2 Hospital District of Helsinki and Uusimaa, Hyvinkää Hospital Area, Kellokoski Hospital, Tuusula, Finland; 3 Vanha Vaasa Hospital, Vaasa, Finland; 4 Department of Psychiatry, Hospital District of Helsinki and Uusimaa, Helsinki University Central Hospital, Helsinki, Finland; 5 Faculty of Medicine, University of Turku, Turku, Finland; 6 Hospital District of Satakunta, Pori, Finland; 7 Turku University Hospital, Turku, Finland

**Keywords:** Methodology, Ethics, Coercion, Inpatient, Perception

## Abstract

**Background:**

Despite improvements in psychiatric inpatient care, patient restrictions in psychiatric hospitals are still in use. Studying perceptions among patients who have been secluded or physically restrained during their hospital stay is challenging. We sought to review the methodological and ethical challenges in qualitative and quantitative studies aiming to describe patients’ perceptions of coercive measures, especially seclusion and physical restraints during their hospital stay.

**Methods:**

Systematic mixed studies review was the study method. Studies reporting patients’ perceptions of coercive measures, especially seclusion and physical restraints during hospital stay were included. Methodological issues such as study design, data collection and recruitment process, participants, sampling, patient refusal or non-participation, and ethical issues such as informed consent process, and approval were synthesized systematically. Electronic searches of CINALH, MEDLINE, PsychINFO and The Cochrane Library (1976-2012) were carried out.

**Results:**

Out of 846 initial citations, 32 studies were included, 14 qualitative and 18 quantitative studies. A variety of methodological approaches were used, although descriptive and explorative designs were used in most cases. Data were mainly collected in qualitative studies by interviews (n = 13) or in quantitative studies by self-report questionnaires (n = 12). The recruitment process was explained in 59% (n = 19) of the studies. In most cases convenience sampling was used, yet five studies used randomization. Patient’s refusal or non-participation was reported in 37% (n = 11) of studies. Of all studies, 56% (n = 18) had reported undergone an ethical review process in an official board or committee. Respondents were informed and consent was requested in 69% studies (n = 22).

**Conclusions:**

The use of different study designs made comparison methodologically challenging. The timing of data collection (considering bias and confounding factors) and the reasons for non-participation of eligible participants are likewise methodological challenges, e.g. recommended flow charts could aid the information. Other challenges identified were the recruitment of large and representative samples. Ethical challenges included requesting participants’ informed consent and respecting ethical procedures.

## Background

Major efforts have been made to reduce the use of coercion in psychiatric care at national and international level [1]. The evidence as to which reduction programme is most successful has been questioned mainly due to a lack of experimental study designs [2-4]. The use of seclusion and restraint (S/R) has been questioned due to a lack of evidence of its safety, usefulness and effectiveness [5,6]. Despite improvements in psychiatric inpatient care, patient restrictions in psychiatric hospitals are still in use. There is also a clear trend towards service-user involvement in treatment decisions, also when the decision concerns coercive measures. Earlier studies have shown that patients tend to view seclusion and restraint as punishment and consider the use of these disempowering measures unnecessary [7-11]. On the other hand, the use of seclusion or restraint may also be perceived to increase feelings of safety and attention paid to inpatients [12,11]. In psychiatric hospital care, patients may face coercion, which may be due to their own disturbed behaviour or condition (agitation, aggression, psychosis) [13]. Forced medication, seclusion and restraint (mechanical or physical) among others are used to treat these situations, and to help patients to avoid hurting themselves or others [2].

Investigating patients’ perceptions of being secluded or restrained [10,14,11], outcome studies [15], or evaluating the effectiveness of S/R includes a number of methodological and ethical challenges [16]. For example, scholars have considered how to avoid additional distress while studying patients who have experienced coercion. Questions include who, when, by what method and how the data should be collected from patients to avoid distressing them [17]. There are also problems with the participant recruitment process as numerous patients refuse to participate in studies [18,19]. Patient recruitment strategies may also be less valid, causing problems in response rates [20]. The main question in recruiting participants with restricted liberty and self-determination is how voluntary participants feel when giving consent to participate in research [21]. The question raised is how a valid informed consent process can be guaranteed [22]. The patient’s ability to absorb information and give consent may be impaired due to her/his condition [23]. Timing in requesting patients’ informed consent is crucial. For example, it is almost impossible to ask patients’ consent before the coercive intervention, which is not predictable [16].

A variety of ethical principles [24-27] and guidelines such as the Declaration of Helsinki 2008 [26], the Singapore Statement on Research Integrity 2010 [27] exist to facilitate high quality research when studying vulnerable patient groups. The Declaration of Helsinki, for example, is the common guideline for research ethics accepted throughout the world and highlights the importance of the proposal process for the research in ethics committees or review boards. Every country has its own legislation, which affects the protocol of broader ethical permission and informed consent procedures [28]. It is unclear how studies focusing on patients’ points of view have taken into account methodological and ethical challenges. Murphy et al. [29] examined methodological challenges in constructing effective treatment for chronic psychiatric patients to make sequential decisions and found that traditional randomized controlled trials (RCT) are not the best option to study adaptive treatment strategies [29]. In a recent review Gupta & Kharawala [21] critically investigated the Informed Consent Procedure (ICD) in psychiatric clinical studies and raised the question of the validity of the consent and the autonomy of the individual subjects [21]. A wide variation in inclusion and exclusion criteria impairs comparability between studies and representativeness. These workers likewise found notable gaps in reporting methodological issues [30].

The purpose of this mixed studies review was to evaluate methodological and ethical challenges in studies investigating coercive methods from the patients’ perspective. Mixed studies reviews integrate qualitative, quantitative, and mixed methods studies [31]. There is a knowledge gap in what researchers should take into account methodologically and research ethically when investigating coercion from patients’ perspective to improve the quality of the studies and to improve the evidence of the care and treatment. To support researchers to conduct studies with vulnerable populations with ethically sensitive topics [23,24,29], we sought to review the methodological and ethical challenges in studies aiming to describe patients’ perceptions of coercive measures, especially seclusion and physical restraints during their hospital stay. We addressed two main questions:

1. What methodological challenges are identified in qualitative and quantitative studies focusing on patients’ perceptions of coercion?

2. What ethical challenges are identified in qualitative and quantitative studies focusing on patients’ perceptions of coercion?

## Methods

### Search strategy

Published research reports were identified using computerized searches of databases: CINAHL (the Cumulative Index of Nursing and Allied Health Literature (1987-2012), Ovid Medline (Medical Literature Analysis and Retrieval System Online, National Library of Medicine, 1976- 2012), and PsychINFO (American Psychological Association, 1982-2012) in April 2012 (Table 1). The search terms in the Cochrane review by Sailas & Fenton [5] on the subject of S/R were used. The search was limited to peer-reviewed reports in English-language journals.

**Table 1:** Databases, search terms and limits for search strategies

**Database and years**	**Search terms**	**Limits**
CINALH (Ebsco) 1987-2012	mental or psychiatr and seclus and mechanical or physical restraint and hospital or inpatient and qualitative or quantitative or rct or empir or random or stud or research or trial and adult	No limits
OvidMedline 1976- 2012	mental or psychiatr and seclus and (mechanical or physical) restraint and qualitative or quantitative or rct or empir or random or stud or research or trial and adult and hospital or inpatient	young adult and adult (19-24 and 19-44) or middle age (45 to 64 years)
PsychINFo 1982-2012	mental or psychiatr and seclus and (mechanical or physical) restraint and qualitative or quantitative or rct or empir or random or stud or research or trial and adult and hospital or inpatient	young adulthood <age 18 to 29 yrs > or 340 thirties <age 30 to 39 yrs > or 360 middle age

### Inclusion criteria

Studies were included if they focused on psychiatric inpatients aged 18-65 years and had faced coercion, forced medication, seclusion or restraint (mechanical/physical) in psychiatric care. We included studies using different study methods, qualitative, quantitative and mixed methods.

Studies focusing on children, adolescents, or geriatric patients, mental retardation, dementia, eating disorders or seclusion/restraint in somatic disorders or chemical restraint alone were excluded. Further, review articles were excluded. Papers that did not specifically address patients’ perceptions related to coercion were likewise excluded.

### Identification of studies

The first author (PS) assessed all the titles and abstracts retrieved for relevance for inclusion in the review. At that stage only those articles were selected that met the inclusion criteria. For the publications selected full texts were obtained and screened to decide on inclusion or exclusion. Any discrepancies were discussed and resolved together with another author (MV).

### Data extraction

PS independently extracted data from the studies included. These, together with information on authors, country and year of publication were listed chronologically from the oldest to the most recent in Table 2. First the qualitative studies were listed and second the quantitative studies. The articles were read carefully and the following data were gathered separately: methods used, study design, data collection methods, recruitment process, participants, sampling, refusal (patients asked to participate, but refused) and non-participation (eligible, but were not offered the opportunity to participate) as well as the time elapsing from the coercive episode to data collection, and data on ethical procedures such as informed consent and ethical proposal process were extracted to reduce the information so that core information was retained and then the data were synthesized in specific categories [32]. Methodological issues were identified by screening the texts and comparing them to the methodological literature. The method section describes the research design, the sample, measures and data collection, and study procedures [32,33,25]. Research design was either mentioned in studies or interpreted according to the literature. Issues concerning research ethics were based on the principles contained in the Declaration of Helsinki (2008) and focused on the formed consent and proposal process. The research ethical process was identified in the text based on its description. In this study we did not use any critical appraisal tools to ascertain the quality of selected studies but only looked at certain criteria emerging from the literature. No tools were specifically designed to assess the methodological quality in mixed study reviews [31].

**Table 2:** Methodological and ethical concerns of the studies

**Author, year country**	**Design**	**Data collection methods**	**Patient recruitment**	**Participants (population and sample size)**	**Ethical approval**	**Patient informed consent asked**	**Patient refusal**	**Non participation**^₫^
**Qualitative**								
Wadeson et al. 1976 [35] USA	Descriptive study with 1 year follow-up	Observations and discussions	Voluntary participation	Acute, hospitalized schizophrenic patients (N = 62, n = 41 secluded)	Not mentioned	Not mentioned	Not mentioned	Not mentioned
Binder et al. 1983 [36] USA	Explorative study	Semi-structured interviews with open-ended and fixed-choice questions	Recruited by researcher	Acute, hospitalized patients (^^, n = 27)	Not mentioned	Consent asked	3 refused	Not mentioned
Outlaw & Lowery 1994 [37] USA	Descriptive study	Unstructured interview	Recruited by researcher after nurse evaluation	Acute, hospitalized restrained patients* (N = 84, n = 84)	Not mentioned	Verbal consent asked by researcher and witnessed by staff member	Not mentioned	Not mentioned
Johnson 1998 [7] USA	Descriptive study	Unstructured interview	Not explained	Acute, hospitalized, restrained patients (^^n = 10)	Not mentioned	Not mentioned	Not mentioned	Not mentioned
Gallop et al. 1999 [8] Canada	Descriptive study	Semi - structured interview	Voluntary participation, posted by treatment centres	Former hospitalized women (^^n = 10)	Not mentioned	Informed written consent asked by researcher after the interview	Not mentioned	Not mentioned
Meehan et al. 2000 [38] Australia	Descriptive study	Semi - structured interview	Not explained	Acute, hospitalized secluded patients (^^n = 12)	Not mentioned	Not mentioned	Not mentioned	Not mentioned
Hoekstra et al. 2004 [39] Netherlands	Descriptive study	Semi – structured interviews	Not explained	Former hospitalized outpatients (^^n = 8)	Ethics Committee	Informed written consent asked by researcher	One refused	Not mentioned
Holmes et al. 2004 [40] Canada	Descriptive study	Unstructured interviews	Not explained	Acute, hospitalized, psychotic, secluded patients (^^n = 6)	Not mentioned	Informed consent asked by researcher	Not mentioned	Not mentioned
Wynn 2004 Norway	Descriptive study	Unstructured interview	Recruited by researcher	Acute, hospitalized patients (^^n = 12)	Ethics Committee	Informed written consent by researcher	Not mentioned	Not mentioned
Chien et al. 2005 [12] China	Descriptive study	Semi – structured interview with open-ended questions	Recruited by researcher	Acute, hospitalized and first time restraint patients (^^n = 30)	Ethics Committee	Informed written consent by researcher	18 refused	50 non participated
Ryan & Happell 2009 [41] Australia	Action research	Unstructured interviews with open-ended questions	Volunteer, recruited in information session by researcher	Patients with former experience of seclusion (n =4)*	Research and Ethics Committee	Informed consent by researcher	18 refused	Not mentioned
Mayers et al. 2010 [42] South Africa	Descriptive and explorative study	Focus group followed by semi - structured interviews with questionnaire	Not explained	Service users earlier hospitalized (N = 43, n = 43)	Ethics Committee	Informed written consent by researcher	Not mentioned	Not mentioned
Sibitz et al. 2011 [43] Austria	Descriptive study	Semi – structured interviews with open-ended questions	Voluntary participation, provided by written information	Service users earlier hospitalized in stable psychiatric condition (^^n = 15)	Ethics Committee	Informed written consent by researcher	Not mentioned	Not mentioned
Kontio et al. 2012 [44] Finland	Descriptive study	Focus group interviews with open-ended question	Recruited by staff	Acute, hospitalized patients (N = 120, n = 30)	Ethics Committee	Informed written consent by staff	16 refused	27 non participated
**Quantitative**								
Soliday 1985 [45] USA	Cross-sectional survey, descriptive study	Self-reported questionnaire	Not explained	Acute, hospitalized patients* (N = 146, n =86)	Not mentioned	Not mentioned	Not mentioned	Not mentioned
Hamill et al. 1989 [46] USA	Explorative study	Structured interview with questionnaire	Recruited by staff	Acutely psychotic, schizophrenic or schizoaffective patients (N = 26, n = 17)	Not mentioned	Consent asked by staff	9 refused	Not mentioned
Mann et al. 1993 [47] USA	Cross-sectional survey, descriptive study	Self-reported questionnaire	Not explained	Acute, hospitalized patients with various diagnosis on voluntary unit (^^n =50)	Not mentioned	Not mentioned	Not mentioned	Not mentioned
Kennedy et al. 1994 [48] USA	Cross-sectional survey, descriptive study	Structured interview with questionnaire	Recruited by researcher after nurse evaluation	Acute, hospitalized schizophrenic or schizoaffective patients (^^n = 25)	Ethics committee	Informed written consent asked by researcher	2 patients	Not mentioned
Ray et al. 1996 [49] USA	Cross-sectional mail survey, descriptive study	Self-reported structured questionnaire by mail	Voluntary participation	Former hospitalized patients (^^n = 1040)	Not mentioned	Not mentioned	Not mentioned	Not mentioned
Meehan et al. 2004 [10] Australia	Cross-sectional survey, explorative study	Self-reported standardized questionnaire	Recruited by research assistance	Acute hospitalized patients* (^^n = 29)	Ethics Committee	Informed written consent asked by assistance	Not mentioned	Not mentioned
Sorgaard [50] 2004 Norway	Intervention study 5 week baseline and 12 week intervention phase	Self –reported standardized questionnaires	Recruited by staff members before discharge	Acute, hospitalized patients (^^n = 190)	Not mentioned	Informed by staff members	Not mentioned	Not mentioned
Frueh et al. 2005 [13] USA	Cross-sectional survey, descriptive study	Self – reported questionnaires	Recruited by researcher	Randomly selected patients in day hospital programme (N = 156, n = 142)	Review boards approvals	Informed written consent and 25 $paid by researchers	14 refused	Not mentioned
Stolker et al 2006 [51] Netherlands	Explorative study	Thematic interview and self-reported structured questionnaire	Not explained	Acute, hospitalized patients (N = 72, n = 54)	Ethics Committee	Informed written consent was obtained by researcher	Not mentioned	6 non participated
Steinert et al. 2007 [52] Germany	Cross-sectional survey, descriptive study	Self – reported questionnaire	Not explained	Acute, hospitalized schizophrenia patients (N = 173, n = 117)	Not mentioned	Informed consent asked by researcher	Not mentioned	Not mentioned
El-Badri et al. 2008 [53] New Zealand	Cross-sectional survey, descriptive, comparative study	Self-reported questionnaire	Not explained	Randomly selected outpatients (n = 111)*	Not mentioned	Not explained	Not mentioned	Not mentioned
Veltkamp et al. 2008 [54] Netherlands	Exploratory study	Self-reported questionnaire	Recruited by researcher	Acute, hospitalized patients (N = 141, n = 104)	Ethics Committee	Informed written consent	24 refused	38 non participated
Whittington et al. 2009 [55] UK	Cross-sectional survey, exploratory study	Self-reported questionnaire	Recruited by staff	Randomly selected acute hospitalized patients (N = 1361)*	Ethics Committee	Informed written consent obtained by staff	Not mentioned	Not mentioned
Keski-Valkama et al. 2010 [56] Finland	Comparative descriptive follow-up study	Structured interview with questionnaire	Recruited and condition evaluated by staff	Hospitalized forensic and acute, hospitalized patients (N = 154, n = 106) in baseline and (n = 83) in follow-up	Ethics Committee	Informed written consent by staff	48 refused	16 non participated
Kjellin & Wallsten 2010 [57] Sweden	Comparative descriptive follow-up study	Structured interviews with questionnaire	Not explained	Acute, hospitalized involuntary and randomly selected patients (N = 375, n =282) in baseline and in follow-up (n = 233)	Ethics Committee	Not mentioned	93 refused	Not mentioned
Bergk et al. [11] Germany	Randomized controlled trial	Semi-structured interview with questionnaire	Randomly and non randomly selected and recruited by researchers	Acute, hospitalized patients (N = 233, n = 108)	Ethical Review board	Informed written consent by researcher	32 refused	Not mentioned
Currier et al. [58] USA	Comparative explorative follow-up study	Self-reported structured questionnaire	Not explained	Acute, emergency patients (N = 151, n = 67 restrained and n = 84 unrestrained)	Human Subject Review Board	Agreement mentioned	Not mentioned	Not mentioned
Georgieva et al. 2012 [59] Netherlands	Explorative study	Self-reported questionnaire	Not explained	Acute hospitalized, first time admitted patients (N = 161, n = 161)	Institution of Board Directors	Not mentioned	75 refused	Not mentioned

*Both patients and staff were participants.

^^Number of total eligible participants was missing.

^₫^Mentioned non participation due to e.g. quick discharge, exclusion criteria, patients’ condition.

### Data analysis

A synthesis was produced based on the data extracted and by using convergent design; qualitative and quantitative synthesis was made of all study types [31]. The rationale to use both qualitative and quantitative data is to gain a wider picture of methodological and ethical issues in the research field of studies concerning patients’ perceptions. The synthesis was made by analysing and synthesizing the key methodological and ethical elements in each study with the aim of transforming individual findings into new conceptualization and interpretations [32,34].

The studies were interpreted by inspecting the methodological demands for studying psychiatric inpatients who had experienced coercive methods. The resulting synthesis was based on all studies included in the analysis. The studies were both qualitative and quantitative; descriptions of the studies were merged in template format and synthesized in Table 2. The information was then analysed both in quantitative and qualitative format as follows: information on recruitment and data collection processes (who, when and how – classified to voluntary participation, researcher recruitment, staff recruitment or not mentioned), research procedures from an ethical point of view (how patients’ informed consent was requested and if ethical approval was mentioned in qualitative and in quantitative studies), the representativeness of the participants (refusal and non-participation) and possible confounding factors related to study protocols. Qualitative and quantitative studies were analysed separately and then merged in the discussion. Study designs were either mentioned by researchers or interpreted in light of the research questions and methods used in the studies.

## Results

### Search results

**Figure 1:**
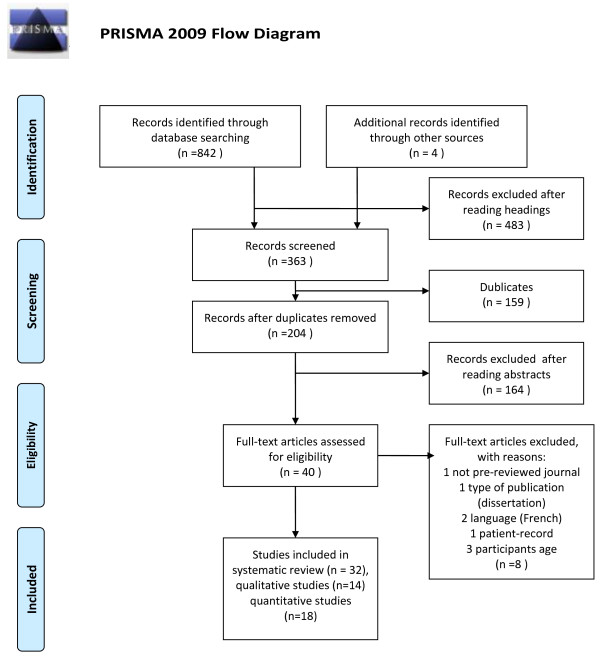
Data selection.

These initial searches resulted in 846 hits: OvidMedline (n = 278), CINALH (n = 157), PsychInfo (n = 407), and four additional records although other sources were found manually. The titles of the studies were reviewed for relevance and 483 were excluded on the basis of inclusion criteria. This left us with 363 items for screening. After removing 156 duplicates, 204 abstracts were carefully reviewed. Based on the inspection, 40 articles published between 1976 and 2012 and focusing on patients’ experiences of coercion were selected. The manuscripts of 40 articles were retrieved and eight were excluded (publication type/forum: dissertation, report published in a non-peer-reviewed journal, and two French-language articles; study design: patient record research and age; children and adolescent included). Thus we eventually included 32 studies, which met all our criteria. A flow chart of data selection is presented in Figure 1.

### Description of the studies

All together 32 articles published in the period 1976–2012 were retrieved, 14 qualitative and 18 quantitative studies (see Table 2). Most of these (n = 22) were published after 2000, nine qualitative studies and 13 quantitative studies. These studies had been conducted in thirteen countries. The majority had been published in the USA (n =11, four qualitative and seven quantitative), and the others were published in the Netherlands (n =4, one qualitative and three quantitative), Australia (n =3, two qualitative and one quantitative), Canada (n = 2 qualitative), Finland (n =2, one qualitative and one quantitative), Germany (n =2 quantitative), Norway (n = 2, one qualitative and one quantitative), and Austria (qualitative), China (qualitative), New Zealand (quantitative), South Africa (qualitative, mixed study), Sweden (quantitative, mixed study) and the United Kingdom (quantitative) one article each. See Table 2.

### Methodological issues of the studies

#### Study designs

Out of the 32 studies, 14 were qualitative [7,8,14,12,35-44] and 18 were quantitative [13,10,15,45-59]. Two of the studies were mixed methods studies [42,51]. A mixed methods study [42] was classified as a qualitative study, because the article reported mainly qualitative data and one mixed methods study classified as a quantitative study reporting mainly quantitative results [51].

In qualitative studies designs were mainly descriptive (n = 11), explorative (n = 1), both descriptive and explorative (n = 1) or action research (n = 1). Six of the qualitative studies mentioned theoretical bases; three of them were based on grounded theory [40,14,43], two on hermeneutic theory [7,41] and one on ethnographic theory [8]. A participatory approach was used in one study, meaning that consumer researchers were used as data collectors [42].

In quantitative studies cross-sectional survey and descriptive design (n = 7), explorative studies (n = 4), comparative follow-up (n = 3), cross-sectional survey with explorative design (n = 2), and intervention, RCT study one each.

#### Data collection methods

The data collection methods used in 14 qualitative studies were observation (n = 1), semi-structured interviews with questionnaire (n = 3) semi-structured questionnaire with open-ended questions (n = 3), unstructured interviews (n = 5), focus group interviews (n = 2).

The data collection methods in 18 quantitative studies were semi-structured (n =1) and structured interviews with questionnaire (n = 4), and self-report questionnaires (n = 13). Validated questionnaires were used to investigate the patients’ perceptions of or attitudes towards coercion or perceived trauma in an inpatient setting [46,10,50,13,45,55,57,15], others used questionnaires developed specifically for their studies [47-49,54,56,58]. See Table 2.

#### Recruitment process

The recruitment process was explained in nine of the qualitative studies [35-37,8,14,12,41,43,44], and in ten of the quantitative studies [46,10,48-50,13,54-56,15]. In qualitative study recruitment was accomplished by staff (n = 1) and by researcher (n =4) or voluntary participation (n = 4) inviting participants by mail, information sessions or aided by the outpatient staff. Information was missing or imprecise in five of the qualitative studies [7,38-40,42].

In quantitative studies recruitment was accomplished by staff (n = 4) and by researcher (n = 5). Information was missing or imprecise in eight of the quantitative studies [45,47,51-53,57-59]. Voluntary participation was mentioned in one study. See Table 2.

#### Participants and sampling

Participants were mostly acute, hospitalized patients (n = 24, nine in qualitative and 15 in quantitative studies). Outpatients (former inpatients) were participants in eight studies (five in qualitative and three in quantitative studies). Staff members were participants in five studies and their experiences or attitudes were compared to those of patients [45,37,53,41,55]. Sample sizes in qualitative studies (n = 14) varied from four patients to 84 (mean 24 participants), and in quantitative studies (n = 18) from 17 to 1 361 (mean 230 participants). See Table 2.

The studies mainly used convenience sampling, meaning that participants who met the inclusion criteria were selected from a certain group in a certain context. Five quantitative studies included randomly selected participants. El-Badri et al. [53] selected participants on certain days of the week. Frueh et al. [13] used computer-generated simple random sampling of eligible participants approached by staff. Whittington et al. [55] selected potential participants randomly; staff assessed participants and then a research assistant approached them to request informed consent but the randomization or inclusion criteria were not explained. Kjellin & Wallsten [57] recruited using both consecutive sampling and randomization, only the exclusion criteria were mentioned. There was one RCT [15]. The method used in the stratified randomization was envelope-method were the envelopes were serial numbered on each ward.

#### Patients’ refusal and non-participation

The number of patients refusing to participate was mentioned (n = 13). Out of these in five qualitative studies patients’ refusal was mentioned (min 1 - max 18 refusals) and in nine studies it was not mentioned. The number of non-participants (eligible, but not offered an opportunity to participate) was explained in two qualitative studies. An explanation for non-participation and numbers of individuals at every stage of the study process was supplied in one qualitative study [44].

Out of all quantitative studies, patients’ refusal was mentioned in eight studies (min 2 max 93 refusals). The number of non-participants (eligible, but not offered an opportunity to participate) was explained in three quantitative studies. An explanation for non-participation and numbers of individuals at every stage of the study process was supplied in five quantitative studies [13,56,57,15,59], and in one study a flow diagram was presented [56]. The main reasons for non-participation were patient’s condition, criteria, short stay in hospital (quick discharge) or not offered participation a chance to participate (for example, staff forgot to ask).

#### Time elapsing between coercion and data collection

The time elapsing between the coercion episode and data collection was mentioned (n = 14) and this varied from during the restraint episode [37] to one month after the experience, mean approximately seven days, in eight qualitative studies [36,8,38,40,14,12,44]. The time elapsing between the seclusion experience and data collection was not specified in three qualitative studies [35,7,39]. Reasons for the time elapsing were mentioned in one study [39]. In addition, Gallop et al. [8] collected data approximately five years after the restraint experience.

The time elapsing between the coercion episode and data collection was mentioned in six quantitative studies [46-48,10,51,15]. Reasons for the time elapsing were mentioned in two studies [10,15]. The time elapsing between the seclusion experience and data collection was not specified in one was quantitative study [49].

Most authors mentioned that data collection had been intended quite soon after the episode, but the patient’s condition or other factors influenced the timing of data collection. Studies (n = 13), interested in patients’ attitudes to coercion, preferences regarding treatment methods or traumatic experiences caused by coercion, and participants who had previously been hospitalized or outpatients, and therefore did not report the time elapsing, in three qualitative studies [41-43] and in ten quantitative studies [45,50,13,52-55,57-59].

### Research ethics

Out of all the studies included in this review, 18 (56%) reported having undergone an ethical review process in an official board or committee. Out of these, approval was reported to have been requested in seven qualitative studies. Respondents were informed and consent was requested in 11 qualitative studies. In seven qualitative studies patients gave informed, written consent. In nine qualitative studies consent was requested by a researcher and in one by staff members. In two qualitative studies staff assessment was mentioned before a researcher approached potential participants to request informed consent [37,14]. In four qualitative studies patients had contacted researchers voluntarily [35,8,41,43].

Out of all studies, 11 quantitative studies reported asking approval from an ethics committee. Respondents were informed and consent was requested in 12 quantitative studies. In eight quantitative studies patients gave informed, written consent. In ten quantitative studies consent was requested by a researcher and in four studies by staff members. In two quantitative studies staff assessment was mentioned before a researcher approached potential participants to request informed consent [48,54]. Five quantitative studies mentioned being part of more extensive research projects and some information reported elsewhere was referred to [50,13,51,53,55].

## Discussion

### Methodological challenges in the studies

This systematic review explored the variation in study designs used in researching patients’ perceptions of coercive measures, which made comparison difficult. The study designs were mainly descriptive or explorative, examining a phenomenon or differentiating it from other phenomena [32,30]. Qualitative studies aimed to explore or describe how patients felt about perceived coercion by interviewing patients, using open-ended questions, questionnaires or focus group interviews. Quantitative studies aimed to explore patients’ perceptions of coercion in larger samples by using cross-sectional survey design or comparing results longitudinally. One further experimental design [15] was identified. The study designs described the situation and proposed that a more profound understanding of psychiatric inpatients’ preferences and experiences was needed. Yet there persists a lack of knowledge of the effectiveness of coercive measures. This may explain the conclusion that more experimental research is needed.

Patients’ recruitment process is crucial when estimating the trustworthiness of findings: the aim is to recruit a representative sample of the population and to meet the required sample size [20,17]. Trustworthiness is related to the process of establishing the validity (credibility) and reliability (dependability) of the findings. Trustworthiness also concerns by what criteria of the results can be judged and how applicable the findings are in other setting [33]. In almost half of the studies the description of the recruitment process was inadequate, which raises the question of trustworthiness of the studies. The information on who invited coerced patients to participate is important as to whether patients participated voluntarily and if their autonomy was respected [18,19]. Most research on human participants involves working with staff. Clinicians who are supportive of research are the best guarantors for success and should be identified at the beginning of the procedure [20]. If the staffs of the study wards or units are responsible for recruitment and data collection, there is a danger that many eligible participants may decline to participate due to unknown reasons. Kontio et al. [44] reported that a total of 26% of potential participants declined to participate and assumed that the staff had deliberately omitted to invite these patients. The studies most successful in their recruitment were those in which the study protocol was carried out in a manner that did not delegate the staff’s responsibility for recruitment, participants’ information, asking consent and data collection. Recommendations such as the STROBE (Strengthening the Reporting of Observational Studies in Epidemiology) statement suggest reporting numbers of participants at every stage, giving reasons for non-participation and using flow diagrams. Only one study was identified using a flow chart to show the number of non-participating but eligible participants.

The question of representative sample size in different studies is unclear and is dependent on study design [33]. In the qualitative studies numbers of participants were small, which includes a limitation of transferability (generalizability) of the results and this was mentioned as a limitation in many articles. In the quantitative studies sample sizes were quite large, although no justification for the research sample sizes was given. None of the quantitative studies tested the sample size through power analysis [32]. The wide variation in populations may affect the generalizability of the results and different groups may require different approaches in building trust and aligning the research goals [20].

#### Confounding factors

Confounding factors and bias affect the results so these should be discussed when interpreting results [60]. Few researchers had paid attention to confounding factors: The influence of the time elapsing between coercion and interview; adaptation to coercive methods; expected responses; researchers’ attitudes when explaining results and making conclusions [9,39] or the relationship between the researcher (interviewer) and participants [38,15]. Involuntarily treated respondents may feel less voluntary and try to please if the investigator was, for example, a staff member involved in treatment. This may be situation especially in qualitative studies. The relationship between respondents and researchers was not always clear. Fortunately most studies reported the relationship and used researchers external to the treatment facilities thus eliminating the effect of the relationship to the results [20,27]. Responding anonymously and independently as well returning responses in sealed envelopes may be easier for patients and gives more reliable answers.

Several other factors may have influenced patients’ accounts because of the time elapsing after the episode (S/R): forgetting, psychotic symptoms, patients may even have been afraid to report how they felt. Hoekstra et al. [39] purposely investigated patients whose seclusion room experience had taken place some time ago to learn about patients’ coping processes after the episode. The place where interviews were conducted might also affect how patients responded, if they felt controlled, forced etc. [7,39].

None of the studies included international comparisons of patients’ experiences of coercive methods, although there are indeed studies on how much and what kind of coercive methods are used internationally [61]. Many researchers reported that generalizability was also hampered by cultural specificity. Cultural specificity was reportedly a religious [12] or organizational culture [10,51,59]. One might consider the influence on the interpretation of researchers’ ideological and theoretical perspectives or personal interests or practical knowledge when their overall conclusions are drawn [60].

### Challenges in research ethics

It has already been discovered that ethical considerations are of insufficient quality in studies [62]. This study also soted that the recommendations of the Declaration of Helsinki (2008) were not adhered to in all reports. In half (56%) of the studies included it was reported that research permission was requested from the appropriate ethics committee. There was also a lack of information on how participants were informed, how consent was obtained, if the consent obtained was in written form and if the participant was really aware of the meaning of the study. Hence it remains unclear how voluntary and informed participants were about their rights. This may lead to unreliable responses. However, the standard of research ethics improved in the more recently published studies.

A crucial question in informed consent is, when psychiatric patients in need of seclusion or restraint due to their condition, mainly their psychotic state, are capable of giving consent and are truly competent to understand the participation and by whom the evaluation of their competence has been made [22]. Patients’ competence cannot be underestimated due to their condition, but information should be given in a form that takes into account the patients’ situation, vulnerability and the voluntary nature of the participation.

## Conclusion

We can conclude on the basis of this review that researching coerced patients’ or service users’ perceptions of coercive interventions is challenging. Many studies in this area were descriptive and explorative, while more experimental studies could guarantee the effectiveness of coercive methods could be described as well. More attention should also be paid to ethical questions, proposal procedure and requesting informed consent. Therefore, researchers clearly need training in how to manage ethically sensitive research topics with vulnerable patient populations.

### Strengths and limitations

The strength of this review was that the methodological and ethical challenges of studies on patients’ perceptions of coercion were identified. A wide variation in study design was found, making comparison of results difficult. In research on vulnerable patients, ethical concerns are core factors. The second finding of weakness in research ethics should be paid more attention although ethical aspects were better addressed in later studies.

On the contrary, limitations of this review a many. First, the search terms used were based on a Cochrane review published in 2000, and other search terms could have been used. Second, the search term coercion yielded many publications, and heterogeneous findings. Third, concentrating on patients’ perceptions of seclusion and restraint may have narrowed the findings, and thereby helped synthesis. Fourth, the studies reviewed used both qualitative and quantitative approaches, so the study procedures differed, making methodological synthesis challenging. Despite these limitations this systematic review provides new insights for psychiatric research to take into account.

### Implications

In light of the findings of this review, we recommend that more attention should be paid to the following issues. First, to improve ethics of the studies, guidelines as the Declaration of Helsinki should be followed. Second, more attention should be paid to how the research frame is described to ensure better quality and comparability. Third, to increase understanding of how representative the research results are, it is important to know why some eligible participants were not included. Non-participants may change the results leading to bias, either or qualitative or quantitative studies. Refusing to participate may lead to assumptions of poor quality of information of the study protocol, fear of consequences and distress due to participating. Using research assistants outside the study organisation in the patient recruitment process and data collection may be useful. Fourth, the studies reviewed included many aspects for improvement as reported by patients. These may have implications in clinical practice as follows: 1) more interaction between staff and patients; 2) need for debriefing, knowledge about the reasons for S/R; 3) staff education and training in how to proactively address situations so that seclusion and restraint could be avoided, and 4) patients’ option to retain their own clothing in seclusion, 5) the opportunity to read, and 6) improvement in the comfort of the S/R environment. And fifth, a quality of the study reports should be increased. In this task, guidelines should be followed from research planning to reporting, for example, CONSORT (The Consolidated Standards of Reporting Trials) Statement [63] or STROBE (Strengthening the Reporting of Observational Studies in Epidemiology) [64]. This would ensure that important details in designing, implementing and reporting research study are taken into account in the study.

## Competing interests

The authors declare that they have no competing interests.

## Authors’ contributions

PS designed the study and involved the data collection, analyses and drafting the manuscript. HP, GJ, JK , and MV were involved in the design of the study, drafting the manuscript and critically reviewed the manuscript. MV checked the data and analyses. All authors have read and approved the manuscript.

## Pre-publication history

The pre-publication history for this paper can be accessed here:

http://www.biomedcentral.com/1471-244X/14/162/prepub
